# Identification of pyrvinium pamoate as an anti-tuberculosis agent *in vitro* and *in vivo* by SOSA approach amongst known drugs

**DOI:** 10.1080/22221751.2020.1720527

**Published:** 2020-02-04

**Authors:** Qing Guan, Lingjun Zhan, Zhi-Hao Liu, Qin Pan, Xu-Lin Chen, Zhen Xiao, Chuan Qin, Xiao-Lian Zhang

**Affiliations:** a Hubei Province Key Laboratory of Allergy and Immunology and Allergy Department of Zhongnan Hospital, Department of Immunology, Wuhan University School of Basic Medical Sciences, Wuhan, People’s Republic of China; b Department of Laboratory Medicine, Xiangyang Central Hospital, Affiliated Hospital of Hubei University of Arts and Science, Xiangyang, People’s Republic of China; c Institute of Laboratory Animal Science, Chinese Academy of Medical Sciences (CAMS), Beijing Key Laboratory for Animal Models of Emerging and Reemerging Infectious; Tuberculosis (TB) Center, Chinese Academy of Medical Sciences, Beijing, People’s Republic of China; d Wuhan Institute of Virology, Chinese Academy of Sciences, Wuhan, People’s Republic of China; e State Key Laboratory of Virology, Medical Research Institute Wuhan University School of Medicine, Wuhan, People’s Republic of China

**Keywords:** *Mycobacterium tuberculosis*, *anti-TB drug screening*, *pyrvinium pamoate*, *selective optimization of side activities approach*, multidrug-resistant *M. tb*

## Abstract

Tuberculosis (TB), caused by *Mycobacterium tuberculosis* (*M.tb*) bacteria, is a leading infectious cause of mortality worldwide. The emergence of drug-resistant *M. tb* has made control of TB more difficult. The selective optimization of side activities (SOSA) approach uses old drugs for new pharmacological targets. In the present study by using SOSA approach, we have successfully identified pyrvinium pamoate (PP) which is capable of inhibiting the growth of mycobacteria, including *M. tb* H37Rv, *Mycobacterium smegmatis*, Bacille Calmette-Guérin (BCG), *M. tb* H37Ra, and drug-resistant *M. tb* clinical isolates *in vitro* from 1280 known drugs library. The MIC_99_ of PP, the minimum inhibitory concentration that inhibits more than 99% of *M. tb* H37Rv and the drug-resistant *M. tb* clinical isolates, ranges from 1.55 to 4.8 µg/mL. Importantly, PP could reduce the bacterial colony-forming units (CFUs) in lung, spleen and liver tissues, and effectively inhibit inflammatory response in *M. tb* H37Rv, multidrug-resistant (MDR) *M. tb* and extensively drug-resistant (XDR) *M.tb*-infected mice. Our results clearly show that the PP has the potential application for treatment of TB.

## Introduction

*Mycobacterium tuberculosis* (*M. tb*), a causative pathogen of tuberculosis (TB), is one of the most dangerous bacteria in the world [1]. TB is the world's major public health problem with high morbidity and mortality [2]. One-third of the world's people are infected with *M. tb*, 8–9 million people suffering from TB each year, and approximately 1.2 million people died from TB in 2018 [2]. *M. tb* strains mainly infect lung, but also infect many other organs such as kidney, brain, spine, lymph nodes, causing extrapulmonary TB [3]. First-line treatments of TB consist of isoniazid (INH), rifampicin (RIF), pyrazinamide (PZA) and ethambutol (EMB) during the first 2 months, continuing with INH and RIF for another 4 months [4]. However, the emergence of multidrug-resistant (MDR) *M.tb* and extensively drug-resistant (XDR) *M.tb* makes the control of TB more difficult. MDR-TB is defined as TB resistant to at least INH and RIF. XDR-TB is defined as MDR-TB plus resistance to any fluoroquinolone and at least one of three injectable drugs (kanamycin, amikacin and capreomycin). Because of limited treatment options for MDR-TB and XDR-TB, they pose additional challenges for global TB control efforts. It is urgent to search for new drugs for treatment of TB, including MDR-TB and XDR-TB.

Because high cost and long approval process limits the clinical application of new anti-TB drugs, the selective optimization of side activities (SOSA) was developed as an innovative strategy to solve these problems. Using the SOSA approach, new potential pharmacological targets of old approved drugs may be identified [5,6]. The safety and pharmacokinetic properties of these old drugs have been well evaluated in humans and animals, so the new biological activities of these approved drugs can be more rapidly and easily applied in clinical practice than new drugs. SOSA has been used in the development of new anti-malarial, antibacterial and antiviral drugs [7-11]. In the present study, we used the SOSA approach to screen out a new anti-*M. tb* drug, pyrvinium pamoate (PP), a US Food and Drug Administration (FDA)-approved drug from the MicroSource (Gaylordsville, CT) library consisting of 1280 drugs and compounds. We have identified that PP is capable of inhibiting multiple mycobacterial species (including *M. tb* H37Rv, H37Ra, BCG and drug-resistant *M. tb* clinical isolates) growth *in vitro* and can protect mice against *M. tb* H37Rv, MDR *M. tb* and XDR *M.tb* infection.

## Materials and methods

### Bacteria, cells and medium

*Mycobacterium smegmatis* (*M. smegmatis*, strain ATCC 70084), *M. bovis* BCG (strain ATCC 35734) and *M. tb* H37Rv (strain ATCC 93009) were from Animal Experimental Center of Wuhan University or China National Institute for the Control of Pharmaceutical and Biological Products [12]. *M. tb* H37Ra strain was kindly provided by Prof. Xiong-Lin Fan (Huazhong University of Science and Technology, Wuhan, China). MDR *M. tb* strain 94789 and XDR *M. tb* strain 8462 were provided by China Food and Drug Administration Institute. The MDR *M. tb* strain 94789 is resistant to INH and RIF. The XDR *M. tb* strain 8462 is resistant to INH, RIF, ofloxacin, and streptomycin (STR). Drug-sensitive *M.tb* clinical isolates and drug-resistant *M. tb* clinical isolates were provided by Wuhan Medical Treatment Center. *M. smegmatis* was cultured in Luria–Bertani (LB) medium with 0.05% Tween 80 or onto Middlebrook 7H10 solid growth medium (BD Biosciences, NJ, USA). Other mycobacterial strains were grown in Middlebrook 7H9 (or 7H10) broth (BD Biosciences, NJ, USA) supplemented with 10% oleic acid-albumin-dextrose-catalase (OADC, BD Biosciences, NJ, USA) and 0.05% Tween 80.

Murine macrophage RAW264.7 cells were purchased from China Center for Type Culture Collection (CCTCC). To prepare murine peritoneal macrophages, thioglycolate-elicited macrophages were prepared by injecting mice with 3.5 mL of 3% sterile thioglycolate media (BD Biosciences, NJ, USA) [12,13]. After 5 days, peritoneal cells were collected by lavage. The cells were cultured overnight and non-adherent cells were removed. The adherent macrophages were collected and stained with anti-F4/80 antibody for purity analysis. F4/80^+^ macrophages were greater than 90% of total cells (data not shown). The cells were cultured in DMEM supplemented with 10% FBS, 100 U/mL penicillin and 100 μg/mL STR.

### Antibiotic and chemicals

The drugs and chemical compounds applied in the library screening were kindly provided by Prof. Xu-Lin Chen (Wuhan Institute of Virology, Chinese Academy of Sciences, China). The library (MicroSource Discovery Systems, Inc. Gaylordsville, CT, USA) consists of 1280 drugs and bioactive compounds. Most of these drugs in this library are FDA-approved, but all have known biological activity. Some drugs, which have not received FDA approval, are approved for clinical use in other countries [14]. These 1280 drugs and bioactive compounds were added to 96 cell culture plates (16 plates of 80 each.) using their laboratory instruments (Echo Sonic Micropipical System).

RIF (CAS#54-85-3), meclizine hydrochloride (CAS#71-58-9), adiphenine hydrochloride (CAS#50-42-0) and BZK (CAS#8001-54-5) were purchased from Wuhan Yi Tai Technology, China. Suloctidil (CAS#54063-56-8) and meclizine hydrochloride (CAS# 31884-77-2) were purchased from Shanghai Tao Su Biochemical Technology Co. Ltd. China. Teniposide (CAS#29767-20-2), isoproterenol hydrochloride (CAS#51-30-9) and doxorubicin (CAS#23214-92-8) were purchased from Shanghai Han Hong Biological Technology Co. Ltd. China. Pyrvinium pamoate (CAS#3546-41-6) was purchased from Dalian Meilun Biotechnology Co. Ltd, China.

### Screening

During the library screening, *M. smegmatis* was diluted to 2.5 × 10^6^ colony-forming units (CFUs)/mL and 200 μL of bacteria solution were added to 96-well cell culture plates in the presence of 25 μM of each candidate drugs and compounds from the library. After 2-day culture, the A_600_ values of bacterial solutions were measured by a microplate reader (SpectraMax® i3, Molecular Devices, LLC, CA, USA). To further confirm the inhibitory effects of selected candidate drugs PP, benzalkonium chloride (BZK), meclizine hydrochloride, suloctidil, doxorubicin, teniposide, isoproterenol hydrochloride, medroxyprogesterone acetate and adiphenine hydrochloride on *M. smegmatis* growth, the bacteria were cultured in the presence of various concentrations of the selected candidate drugs (0–10 μg/mL), and A_600_ values and CFUs of *M. smegmatis* were determined.

To assess the effects of the candidate drugs on growth of BCG and *M. tb* H37Ra, these mycobacteria were diluted to 2.5 × 10^6^ CFUs/mL in 4 mL of 7H9 liquid medium containing various concentrations of PP, BZK, meclizine hydrochloride, suloctidil, doxorubicin and teniposide respectively (0–10 μg/mL). To assess the effects of PP on the growth of *M. tb* H37Rv, *M. tb* H37Rv was diluted to 2.5 × 10^6^ CFUs/mL in 4 mL of 7H9 liquid medium containing various concentrations of PP. After 30-day culture, the A_600_ values of bacterial solutions were measured. In positive control group, *M. tb* H37Rv was cultured without the drugs. 7H9 liquid medium was used as medium control. Inhibition % = (A_600__experimental group−_A_600__medium control_)/(A_600 positive control−_A_600__medium control_). The half maximal inhibitory concentration (IC_50_) values were calculated using GraphPad 6.0 software (Graphpad software, San Diego, CA). The MIC_99_ values were calculated using the statistical package IBM SPSS Statistics software (SPSS) (IBM Corp., Armonk, NY, USA).

To assess the effects of PP on the growth of drug-sensitive *M. tb* and drug-resistant *M. tb* clinical isolates, these clinical isolates were diluted to 2.5 × 10^6^ CFUs/mL in 4 mL of 7H9 liquid medium containing various concentrations of PP (0–10 μg/mL). After 15-day culture, the A_600_ values of bacterial solutions were measured by Maxwell concentration detector in Wuhan Medical Treatment Center.

### Cell viability analysis

Cell viability analysis was performed by CCK-8 assay according to the manufacturer's recommendations (Beyotime, Shanghai, China). Murine peritoneal macrophages and RAW264.7 cells were seeded in 96-well plates (2 × 10^4^ cells/100 μL/well). The cells were cultured in the presence of various concentrations of PP (0.1–10 μg/mL) for 24 h. CCK-8 solution (10 μL/well, Beyotime, Shanghai, China, #C0038) was added to each well. The plates were incubated for 2 h and A_450_ values were measured using a microplate reader. CC_50_ (drug concentration required to reduce cell viability by 50%) of PP was calculated using GraphPad 6.0 software. The selective index (SI) was calculated according to the equation: SI = CC_50_ /IC_50_.

### Drug susceptibility testing (DST) and determination of minimum inhibitory concentration (MIC)

DST was performed using MGIT SIRE kit and liquid *Mycobacterium* Growth Indicator Tube system (MGIT) 960 as previously described [15]. In addition to 7H9 liquid medium, the MGIT tube contains an oxygen-quenched fluorochrome, tris 4,7-diphenyl-1, 0-phenonthroline ruthenium chloride pentahydrate, embedded in silicone at the bottom of the tube. During bacterial growth within the MGIT tube, the free oxygen is utilized and the intensity of fluorescence from the tubers is directly proportional to the extent of oxygen depletion. Growth of mycobacteria increases the fluorescence. The assessment of drug susceptibility was determined automatically at the point at which the proportional-growth control reached 400 growth units (GU). The instrument declares a tube negative if it remains negative for six weeks (42 days). In the current study, *M. tb* H37Rv, MDR *M. tb* strain 94789 and XDR *M. tb* strain 8462 were analyzed by DST. These mycobacterial strains were cultured in the presence of INH, RIF or STR. The recommended concentrations were 0.2 μg/mL for INH, 5 μg/mL for RIF and 1 μg/mL for STR.

To determine the MICs of PP against different *M. tb* strains, *M. tb* H37Rv, MDR *M. tb* 94789 and XDR *M. tb* 8462 were cultured in the various concentrations of PP (0.1–3 μg/mL). By the use of the automated Bactec MGIT 960 system, all strains were interpreted objectively and the MICs were determined. The lowest concentration of drug that inhibits growth of more than 99 % of the bacterial population was considered to be the MIC_99_.

### Murine TB infection models

Female C57BL/6 mice (6–8 week, 17∼19 g, SPF) were purchased from Beijing Charles River Laboratories. The use of these laboratory animals has been approved by the Committee on the Use and Management of Laboratory Animals, Institute of Medical Laboratory Animals, Chinese Academy of Medical Sciences, Beijing (Approval No. ZLJ17002). Animal rearing and experimentation were carried out in the Grade 3 Laboratory of Biosafety, Institute of Medical Laboratory Animals, Chinese Academy of Medical Sciences (ABSL3-059).

Totally 96 female C57BL/6 mice were used and six mice were used for each group. The mice were infected (*i. v.*) with *M. tb* H37Rv (1 × 10^6^ CFUs/mouse), *M. tb* H37Rv 94789 (1 × 10^4^ CFUs/mouse) or *M. tb* H37Rv 8462 (1 × 10^5^ CFUs/mouse) on week −1. After a week of infection (in Week 0), the mice were treated with RIF (20 mg/kg/day, 5 days/week), EMB (100 mg/kg/day, 5 days/week) or PP (0.5 mg/kg/day in Week 1, 1 mg/kg/day in Week 2–5 and 1.5 mg/kg/day in Week 6, 5 days/week). In EMB + PP combination therapy group, the mice were treated with EMB (100 mg/kg/day, 5 days/week) and PP (0.5 mg/kg/day in Week 1, 1 mg/kg/day in Week 2–5 and 1.5 mg/kg/day in Week 6, 5 days/week). In RIF + PP combination therapy group, the mice were treated with RIF (20 mg/kg/day, 5 days/week) and PP (0.5 mg/kg/day in Week 1, 1 mg/kg/day in Week 2–5 and 1.5 mg/kg/day in Week 6, 5 days/week) PP was administered by intraperitoneal injection, while other drugs were administered by oral gavage. After 4 weeks or 6 weeks of therapy treatment, the *M. tb* loads in the lungs, spleens and livers mice were assessed. The sections of lungs, spleens and livers were fixed in 4% paraformaldehyde and pathological sections were examined with hematoxylin–eosin (H&E) staining.

### Statistical analysis

Data were analyzed with SPSS software and GraphPad 6.0 software. Differences were considered to be statistically significant for *P*-values <0.05. Statistical significance was determined by Student’s *t*-test or one-way ANOVA followed by Newman–Keuls post hoc test.

## Results

### Six drugs were selected from a library of 1280 candidates for inhibiting *M. smegmatis* growth *in vitro*

**Figure 1.**
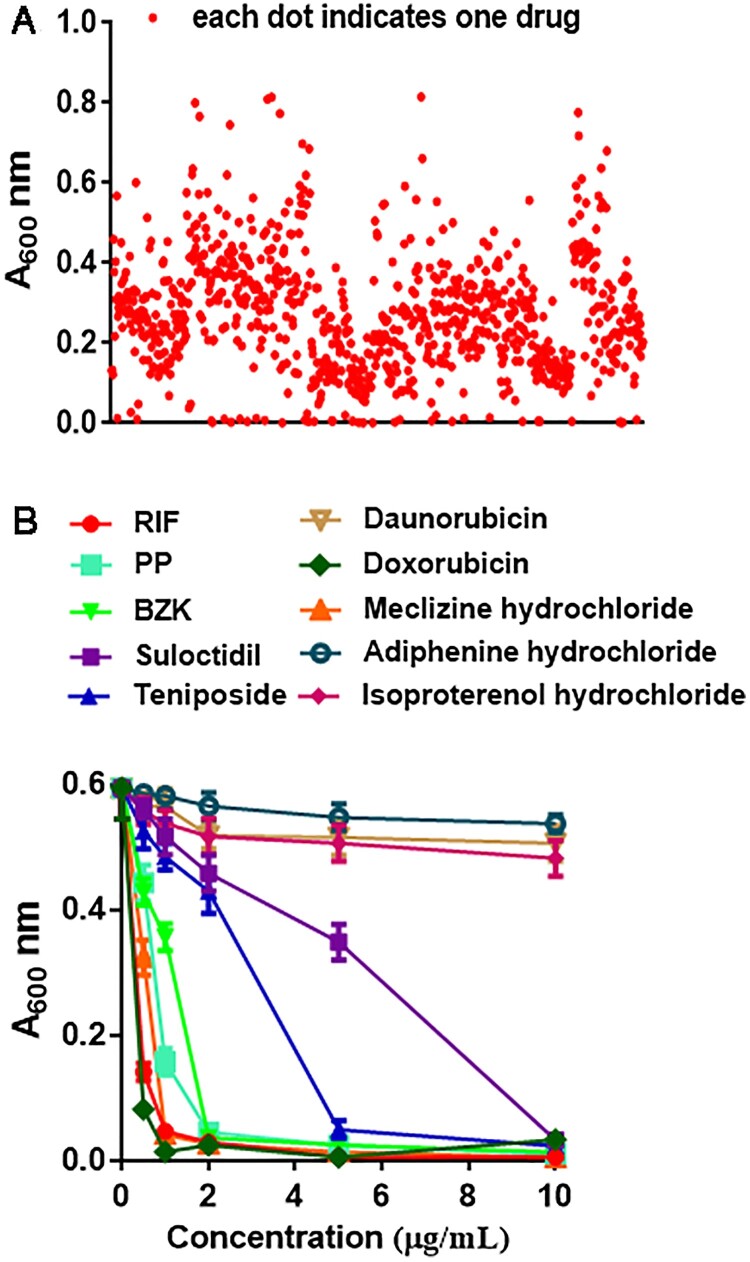
Six drugs are screened out from 1280 candidate drugs for inhibiting *M. smegmatis* growth *in vitro*. (A) A_600_ values of *M. smegmatis* cultured in the presence of 1280 candidate drugs. The bacteria were cultured in the 7H9 liquid medium containing 25 μM compounds in 96-well cell culture plate. After 48 h culture, the bacterial growth was determined by A_600_ value. (B) A_600_ values of *M. smegmatis* cultured in the presence of various concentrations of the indicated drugs. After 48 h culture, the bacterial growth was determined by A_600_ value. The data in (B) are shown as the means ± SD (*n*=3).

*M. smegmatis* has been commonly used to screen new anti-TB drugs due to its rapid growth rate [16]. In the current study, to find a new anti-*M. tb* drug, *M. smegmatis* was firstly used as a target for screening out *M. tb* inhibitors from the 1280 candidate drugs and compounds. *M. smegmatis* was cultured in liquid medium containing 25 μM of each candidate drug, and A_600_ value of *M. smegmatis* was determined to evaluate effects of candidate drugs on bacterial growth (Figure 1(A)). 67 drugs out of the 1280 candidate drugs were shown to inhibit *M. smegmatis* growth. We removed the anti-*M.tb* drugs (including INH, RIF, etc) and toxic compounds (including acetic acid phenyl mercury, thioglycans, etc) from these 67 drugs, and chose nine drugs (Table 1, Supplemental Table 1), including PP, benzalkonium chloride (BZK), meclizine hydrochloride, suloctidil, doxorubicin, teniposide, isoproterenol hydrochloride, medroxyprogesterone acetate and adiphenine hydrochloride, that would be employed in the next round of screen.

**Table 1.** The MIC_99_ values of ten drugs on *M. smegmatis*.

Drugs	MIC_99_ (μg/mL)
RIF	5
Doxorubicin	5
Pyrvinium pamoate (PP)	9
Meclizine hydrochloride	10
Benzalkonium chloride (BZK)	>10
Suloctidil	>10
Teniposide	>10
Adiphenine hydrochloride	>10
Daunorubicin	>10
Isoproterenol hydrochloride	>10

To further confirm the inhibitory effects of these candidate drugs, *M. smegmatis* was cultured in liquid medium containing various concentrations of the drugs (0–10 μg/mL), and A_600_ value of *M. smegmatis* was determined. Adiphenine hydrochloride, daunorubicin and isoproterenol hydrochloride, have limited inhibitory effects on *M smegmatis* growth (Figure 1(B)). Six drugs, PP, BZK, meclizine hydrochloride, suloctidil, doxorubicin and teniposide, were shown to inhibit *M. smegmatis* in dose-dependent manner (Figure 1(B)). PP and doxorubicin showed the strongest inhibitory effects on *M. smegmatis* growth. The MIC_99_ values of PP and doxorubicin on *M. smegmatis* were 9 and 5 μg/mL, respectively (Table 1).

### PP inhibits standard laboratory strain *M. tb* H37Rv growth *in vitro*

**Figure 2.**
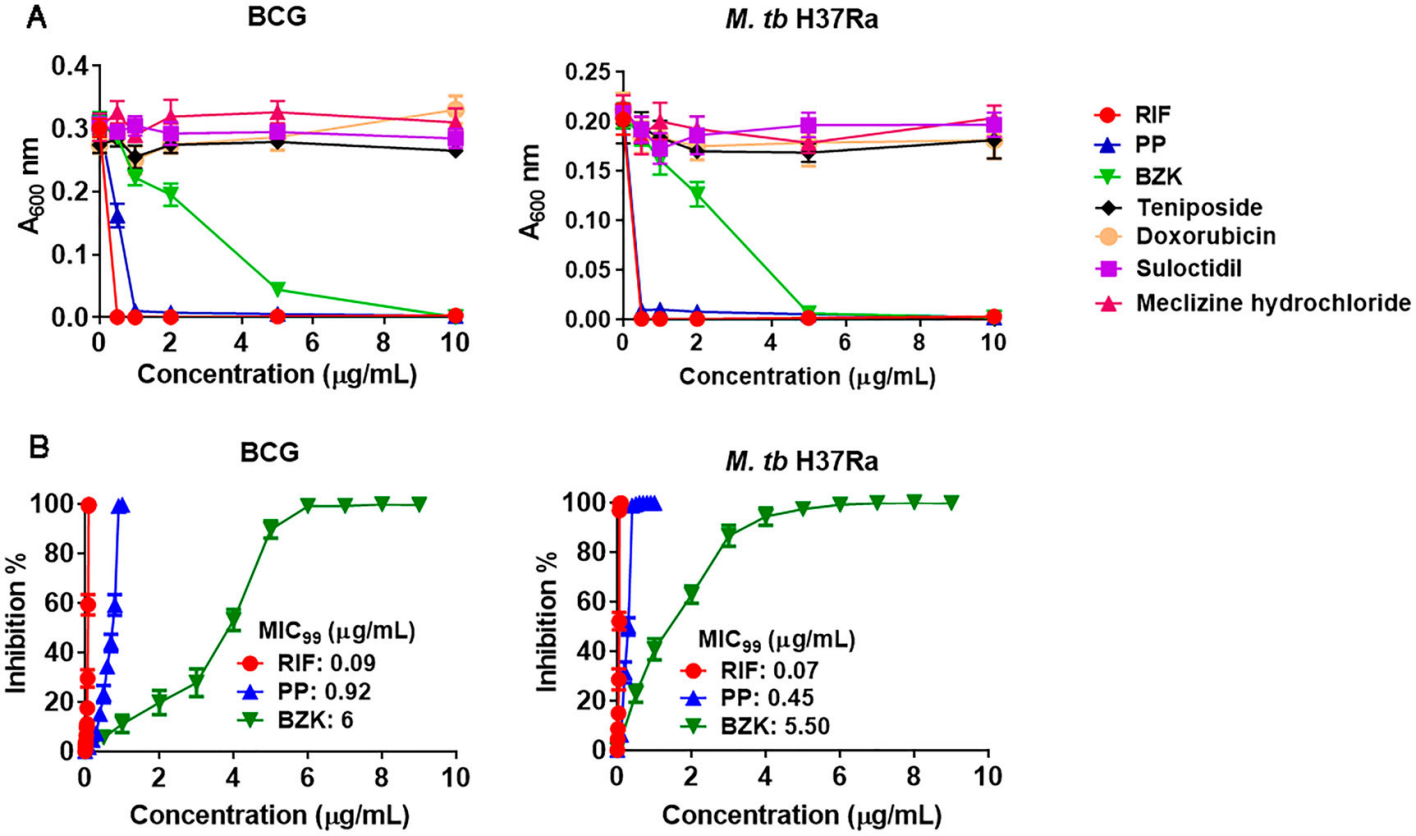
PP inhibits the growth of BCG and *M. tb* H37Ra *in vitro*. (A) and (B) PP and BZK inhibited the growth of BCG and *M. tb* H37Ra. BCG and *M. tb* H37Ra were cultured in the presence of various concentrations of the drugs as indicated for 30 days, and A_600_ values of BCG and *M. tb* H37Ra were determined. In the infection control group, *M. tb* H37Rv was cultured without the drugs. 7H9 liquid medium was used as medium control. (A) A_600_ values of BCG and *M. tb* H37Ra. (B) Inhibition % of BCG and *M. tb* H37Ra by PP and BZK, and MIC_99_ of PP and BZK. Inhibition %= (A_600 experimental group−_A_600 medium control_)/(A_600 positive control−_A_600 medium control_). All data are shown as the means ± SD (*n*=3). IC_50_ values were calculated using GraphPad 6.0 software. The MIC_99_ values were calculated using the statistical package IBM SPSS Statistics software.

We then assessed the activity of these drugs on growth of BCG, *M. tb* H37Ra and H37Rv. BCG, Bacille Calmette-Guérin, is a live, attenuated vaccine derived from *Mycobacterium bovis*, and *M. tb* H37Ra is an attenuated strain originated from *M. tb*. Both of these *Mycobacteria* are more closely related to the virulent strain *M. tb* H37Rv than *M. smegmatis*. BCG and *M. tb* H37Ra were cultured in the presence of various concentrations of the drugs for 30 days. As shown in Figure 2(A), only two drugs, PP and BZK, inhibited the growth of BCG and *M. tb* H37Ra in a dose-dependent manner, and other four drugs had no inhibitory effect on the growth of BCG and *M. tb* H37Ra.

To assess the inhibitory effects of PP and BZK on BCG and *M. tb* H37Ra, the inhibition % of mycobacterial growth and the MIC_99_ values for PP and BZK were calculated (Figure 2(B) and Table 2). Both PP and BZK significantly inhibited BCG and *M. tb* H37Ra growth in the comparative low concentrations (Figure 2(B) and Table 2). However, BZK showed less effective than RIF, and PP was similar effectiveness as compared to RIF (Figures 2(A,B) and Table 2). The MIC_99_ values of PP on BCG and *M. tb* H37Ra were 0.92 and 0.45 μg/mL, respectively, which were much lower than those of BZK (6 μg/mL on BCG and 5.5 μg/mL on *M. tb* H37Ra, respectively, Figure 2(B) and Table 2). Therefore, PP was selected and its inhibitory effect on virulent *M.tb* was evaluated in the following experiments.

**Table 2.** The MIC_99_ values of the selected drugs on BCG and H37Ra.

Drugs	MIC_99_ (μg/mL)	
	BCG	H37Ra
RIF	0.2	0.3
PP	0.92	0.45
BZK	6	5.5
Meclizine hydrochloride	>10	>10
Suloctidil	>10	>10
Doxorubicin	>10	>10
Teniposide	>10	>10

**Figure 3.**
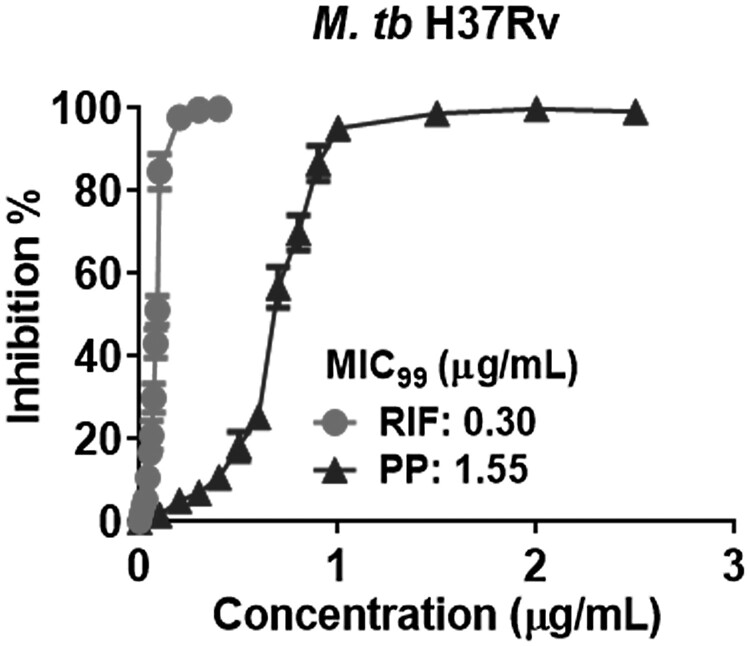
PP inhibits the standard laboratory strain *M. tb* H37Rv growth *in vitro*. *M. tb* H37Rv was cultured in the presence of various concentrations of PP for 30 days, and the A_600_ value of *M. tb* H37Rv was measured, Inhibition % and MIC_99_ were calculated. The data are shown as the means ± SD (*n* = 3).

To assess the inhibitory effects of PP on standard laboratory strain *M. tb* H37Rv, *M. tb* H37Rv was cultured in the presence of various concentrations of PP for 30 days, and the inhibition % of *M. tb* H37Rv growth and PP MIC_99_ value were calculated (Figure 3). PP was capable of inhibiting *M. tb* H37Rv growth in a dose-dependent manner, although PP showed a little less effective than RIF (Figure 3). The MIC_99_ values were 1.65 μg/mL for PP and 0.30 μg/mL for RIF on *M. tb* H37Rv. Therefore, PP has an inhibitory effect on *M. tb* H37Rv growth *in vitro*.

SI value reflects the “fitting” of the drug to the particular target [14]. The higher SI value is, the more suitable the drug for the particular target is. To further evaluate PP effects on various *M. tb* strains, we calculated the SI values of PP for laboratory strain *M. tb* and clinical *M. tb* isolates. As shown in Table 3, the SI values of PP for laboratory standard strain *M. tb* H37Rv were 3.69, which was lower than that SI values for most of clinical isolates *M. tb* (3.40-6.09). These results suggested that PP “fits” more selectively to clinical isolates than laboratory strain *M. tb* H37Rv.

**Table 3.** SI and MIC_99_ values of PP on Mycobacteria.

Bacteria	IC_50_ (μg/mL)	CC_50_ (μg/mL)	SI (CC_50_/IC_50)_	MIC_99_ (μg/mL)
BCG	0.75	2.4	3.2	0.92
*M. tb* H37Ra	0.3	2.4	8	0.45
*M. tb* H37Rv	0.65	2.4	3.69	1.55
**Drug-sensitive *M. tb***				
Strain 1	0.60	2.4	4.03	4.8
Strain 2	0.71	2.4	3.4	1.9
Strain 3	0.47	2.4	5.1	4.0
Strain 4	0.43	2.4	5.6	4.5
Strain 5	0.46	2.4	5.2	4.6
**MDR *M. tb***				
Strain 1	0.45	2.4	5.35	4.5
Strain 2	0.39	2.4	6.09	1.9
Strain 3	0.51	2.4	4.66	2.0
Strain 4	0.39	2.4	6.09	4.8
Strain 5	0.71	2.4	4.37	4.6
**INH-resistant *M. tb***				
Strain 1	0.49	2.4	4.89	3.5
Strain 2	0.50	2.4	4.74	4.0
Strain 3	0.80	2.4	2.98	4.8
Strain 4	0.46	2.4	5.17	4.6
Strain 5	0.51	2.4	4.67	1.8
**RIF-resistant *M. tb***				
Strain 1	1.0	2.4	2.4	4.8
Strain 2	0.74	2.4	3.23	2.0

### PP inhibits growth of both drug-sensitive and drug-resistant *M. tb* clinical isolates *in vitro*

**Figure 4.**
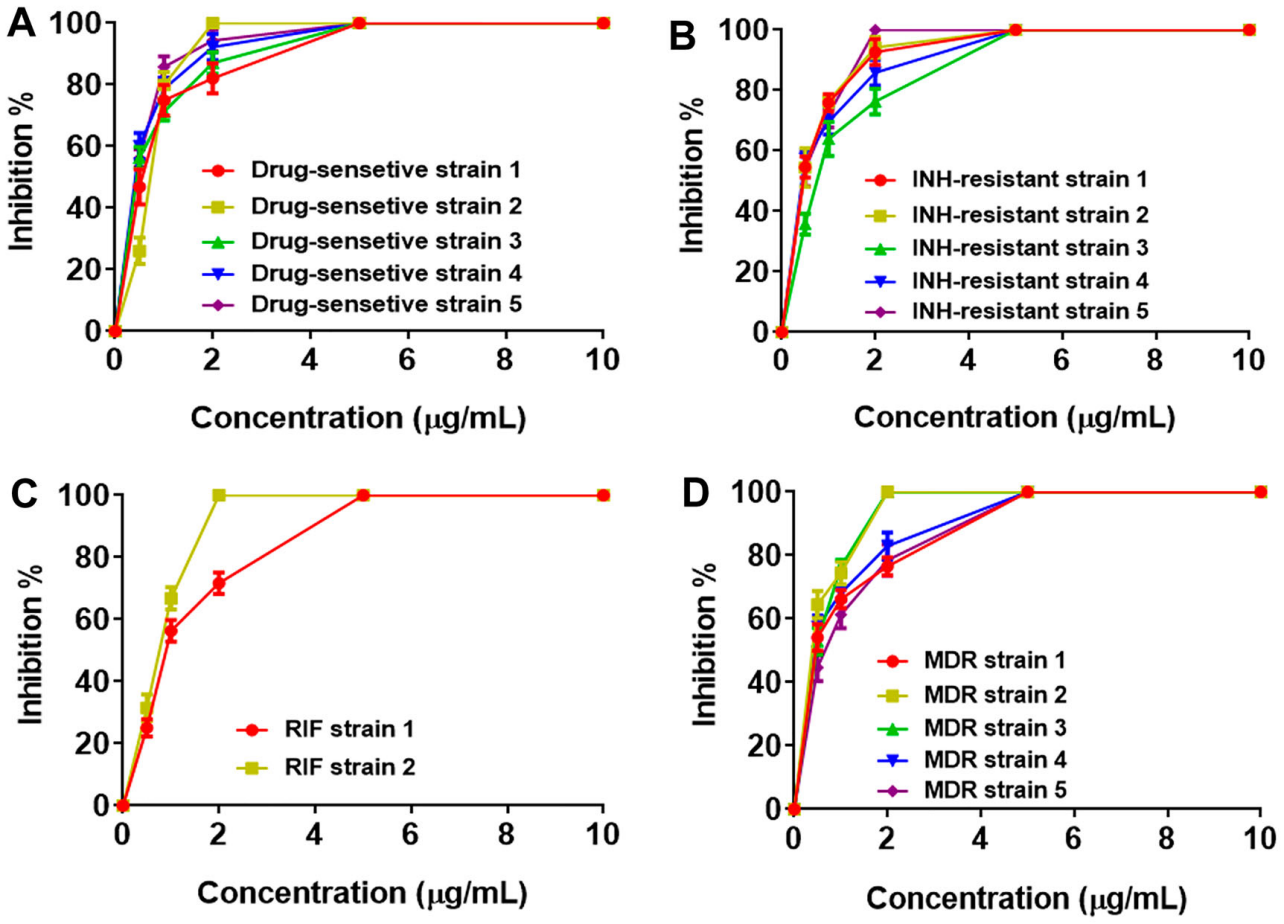
PP inhibits both drug-sensitive and drug-resistant *M. tb* clinical isolates growth *in vitro.* Drug-sensitive and drug-resistant *M. tb* clinical isolates were cultured in the presence of various concentrations of PP for 15 days, and the A_600_ values of *M. tb* clinical isolates were measured. Inhibition % and MIC_99_ were calculated. (A) Inhibition % of drug-sensitive *M. tb* clinical isolates (strain 1–5) and MIC_99_ values of PP; (B) Inhibition % of INH-resistant *M. tb* clinical isolates (strain 1–5) and MIC_99_ values of PP; (C) Inhibition % of RIF-resistant *M. tb* clinical isolates (strain 1 and 2) and MIC_99_ values of PP; (D) Inhibition % of MDR-resistant *M. tb* clinical isolates (strain 1–5) and MIC_99_ values of PP. The data in (A)-(D) are shown as the means ± SD (*n* = 3).

To assess the inhibitory effect of PP on the clinical isolates of *M. tb*, both drug-sensitive and drug-resistant *M. tb* clinical isolates were employed. The MIC_99_ values of PP on five drug-sensitive *M. tb* clinical isolates were 4.8, 1.9, 4.0, 4.5 and 4.6 µg/mL, respectively (Figure 4(A) and Table 3). The MIC_99_ values of PP on five INH -resistant *M. tb* clinical isolates were 3.5, 4.0, 4.8, 4.6 and 1.8 µg/mL, respectively (Figure 4(B) and Table 3). The MIC_99_ values of PP on two RIF-resistant *M. tb* clinical isolates were 4.8 and 2.0 µg/mL (Figure 4(C) and Table 3). The MIC_99_ values of PP on five MDR *M. tb* were 4.5, 1.9, 2.0, 4.8 and 4.6 µg/mL, respectively (Figure 4(D) and Table 3). These results strongly demonstrated that PP inhibited growth of both drug-sensitive and drug-resistant *M. tb* clinical isolates.

### PP inhibits *M. tb* h37Rv infection in mice

**Figure 5.**
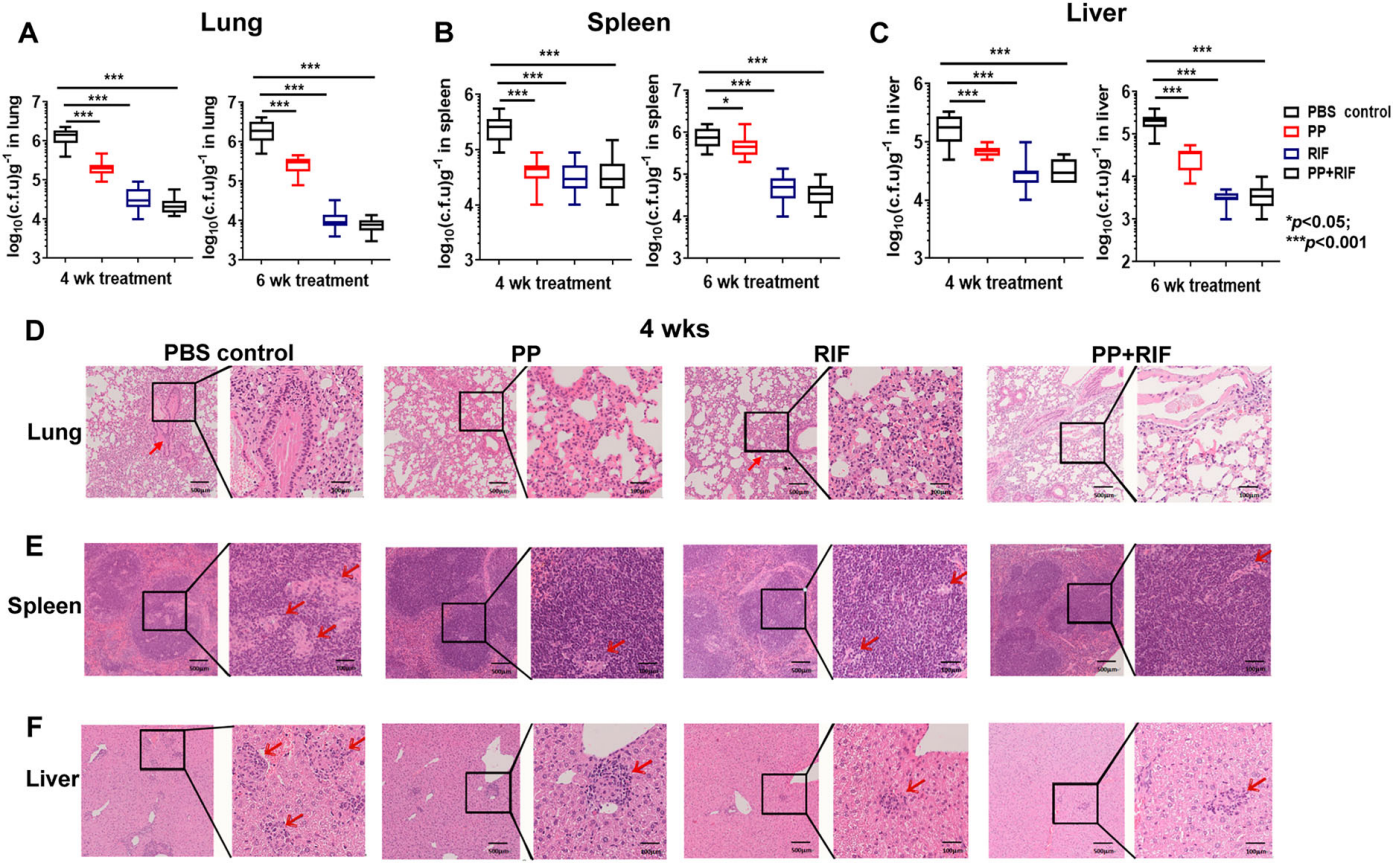
PP inhibits *M. tb* H37Rv growth in mice. The mice were infected (*i. v.*) with *M. tb* H37Rv (1 × 10^6^ CFU/mouse), and subjected to treatment with PP, RIF or PP in combination with EBM. After 4 weeks or 6 weeks of therapy treatment, the bacterial loads in the lungs (A), spleens (B) and livers (C) were assessed. The pathological sections of lungs (D), spleens (E) and livers (F), obtained 4 weeks after therapy treatment, were examined with H&E and evaluated by light microscopy. The data in (A)–(C) are shown as the means ± SD (*n* = 6). The arrows in (D)–(F) indicate the pulmonary granulomas (D), disorganization of WP and RP in spleen (E) and liver lesions (F).

Next, we assessed the protective effects of PP in a mouse model against *M. tb* infection. The mice were *i.v.* infected with *M. tb* H37Rv on Week −1. These mice were treated with PP, RIF or PP plus RIF weekly from week 0 to week 6. After 4 weeks or 6 weeks of the treatment, as shown in Figure 5(A–C), PP treatment significantly reduced the bacterial CFUs in lung, spleen and liver tissues compared with PBS control group, indicating that PP treatment inhibited *M. tb* H37Rv growth *in vivo* (Figure 5(A–C)). However, PP did not have a more effective antibacterial effect than RIF. PP combined with RIF treatment did not further reduce the CFUs in mice, indicating no synergic effects between PP and RIF (Figure 5(A–C)).

Histological analysis revealed that *M. tb* infection caused severe pathological changes and inflammation response in lung, spleen and liver tissues in the *M. tb* H37Rv group. After treatment with PP, RIF and PP plus RIF, the pulmonary lesion and inflammation alleviated, especially the 6-week treatment with PP markedly and effectively reduced lesion and inflammatory in the lungs compared with the other groups (Figure 5(D–F), Supplemental Figure 1). Granulomas, inflammatory cells infiltration and thickening of bronchial alveolar wall were observed in the lungs in the PBS control group (Figure 5(D)). Disorganization of white pulp (WP) and red pulp (RP) in spleens and hepatocyte lesion were also observed in *M. tb* H37Rv plus PBS control group (Figure 5(E,F)). In PP, RIF and PP plus RIF groups, the WP and RP were clearly evident and lymphoid hyperplasia occurred in spleens, indicating the active proliferation of immune cells after the drug treatment (Figure 5(E)). Compared with the PBS control group, 6-week treatment of PP and PP plus RIF ameliorated the hepatocyte swelling, indicating that PP has a potential advantage of liver protection over RIF (Supplemental Figure 1). These results strongly demonstrated that PP treatment reduced severe pathological changes and inflammatory response, and inhibited *M. tb* H37Rv infection in mice.

### PP inhibits MDR *M.tb* and XDR *M.tb* infection in mice

To assess whether PP had protective effects on drug-resistant *M. tb* infection *in vivo*, the MDR *M. tb* 94789 and XDR *M. tb* 8462 were used. Before mice infection, we determined that the MDR *M. tb* strain 94789 was resistant to INH and RIF, and the XDR *M.tb* strain 8462 was resistant to INH, RIF, ofloxacin, and streptomycin (STR). The results showed that the MIC_99_ of PP against *M. tb* H37Rv, *M. tb* 94789 and *M. tb* 8462 were 1.5 μg/mL, 3 μg/mL, and 0.75 μg/mL, respectively (Supplemental Table 2).

**Figure 6.**
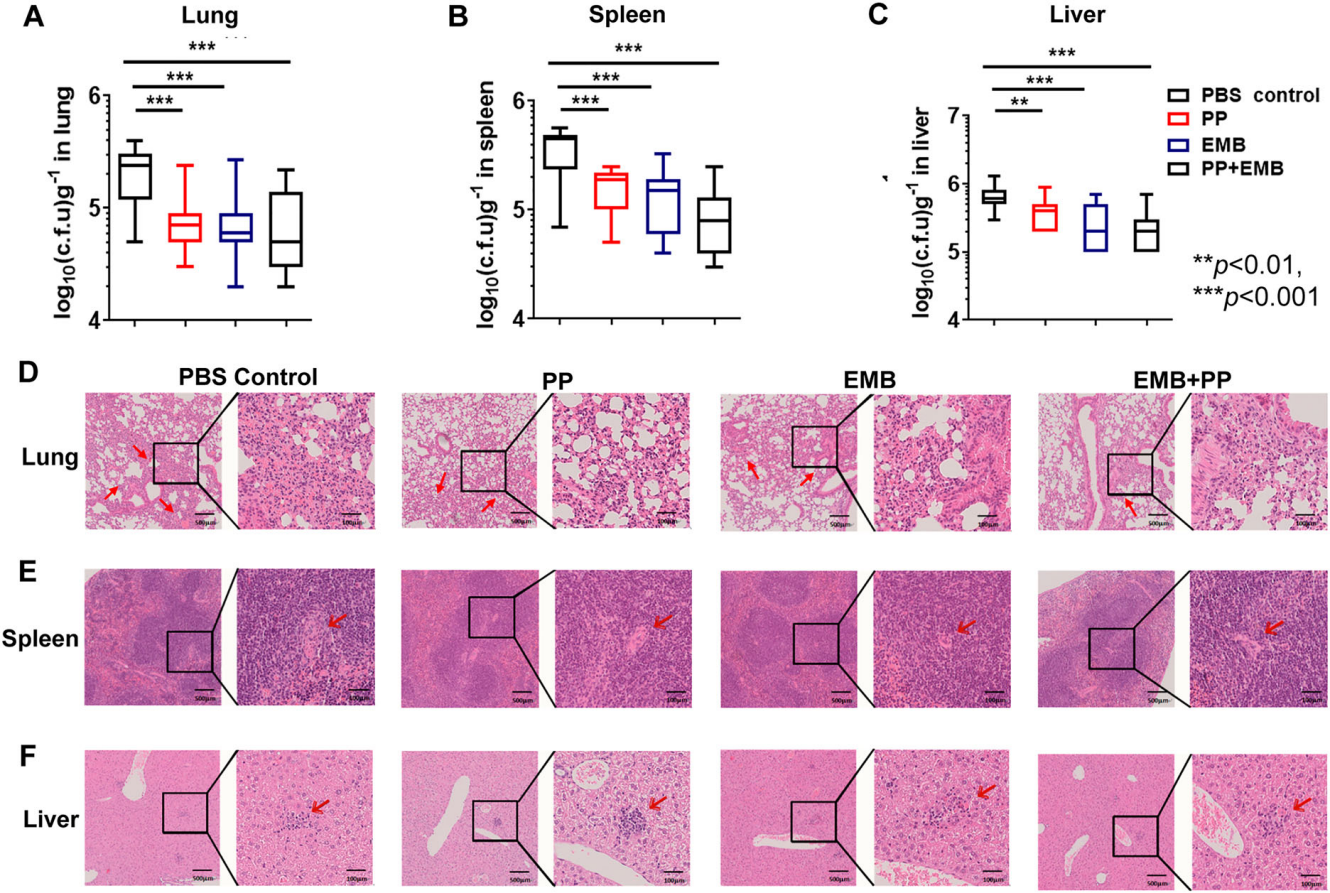
PP inhibits MDR *M. tb* 94789 growth in mice. The mice were infected (*i. v.*) with *M. tb* 94789 (1 × 10^4^ CFU/mouse), and subjected to treatment with PP, EBM or PP in combination with EBM. After 4 weeks of therapy treatment, the bacterial loads in the lungs (A), spleens (B) and livers (C) were assessed. The pathological sections of lungs (D), spleens (E) and livers (F) were examined with H&E and evaluated by light microscopy. The data in (A)–(C) are shown as the means ± SD (*n* = 6). The arrows in (D)-(F) indicate the pulmonary granulomas (D), disorganization of WP and RP in spleen (E), and liver lesions (F).

In the mouse model of *M. tb* 94789 infection, the mice were *i.v.* infected with *M. tb* 94789 on week −1. Because *M. tb* 94789 is RIF resistant, EMB treatment was used as a drug control. The mice were treated with PP, EMB or PP plus EMB on Week 0. After 4 weeks of treatment, both PP or EMB treatment significantly reduced the bacterial CFUs in lung, spleen and liver tissues compared with the PBS control group (Figure 6(A–C)), indicating that PP treatment inhibited MDR *M. tb* 94789 growth *in vivo* (Figure 6(A–C)). However, PP did not have a better antibacterial effect than EMB and there was no synergic effects between PP and EMB. Similarly, histological analysis revealed that PP treatment effectively reduced lesion and inflammation in lung, spleen and liver tissues by *M. tb* 94789 infection (Figure 6(D–F)).

**Figure 7.**
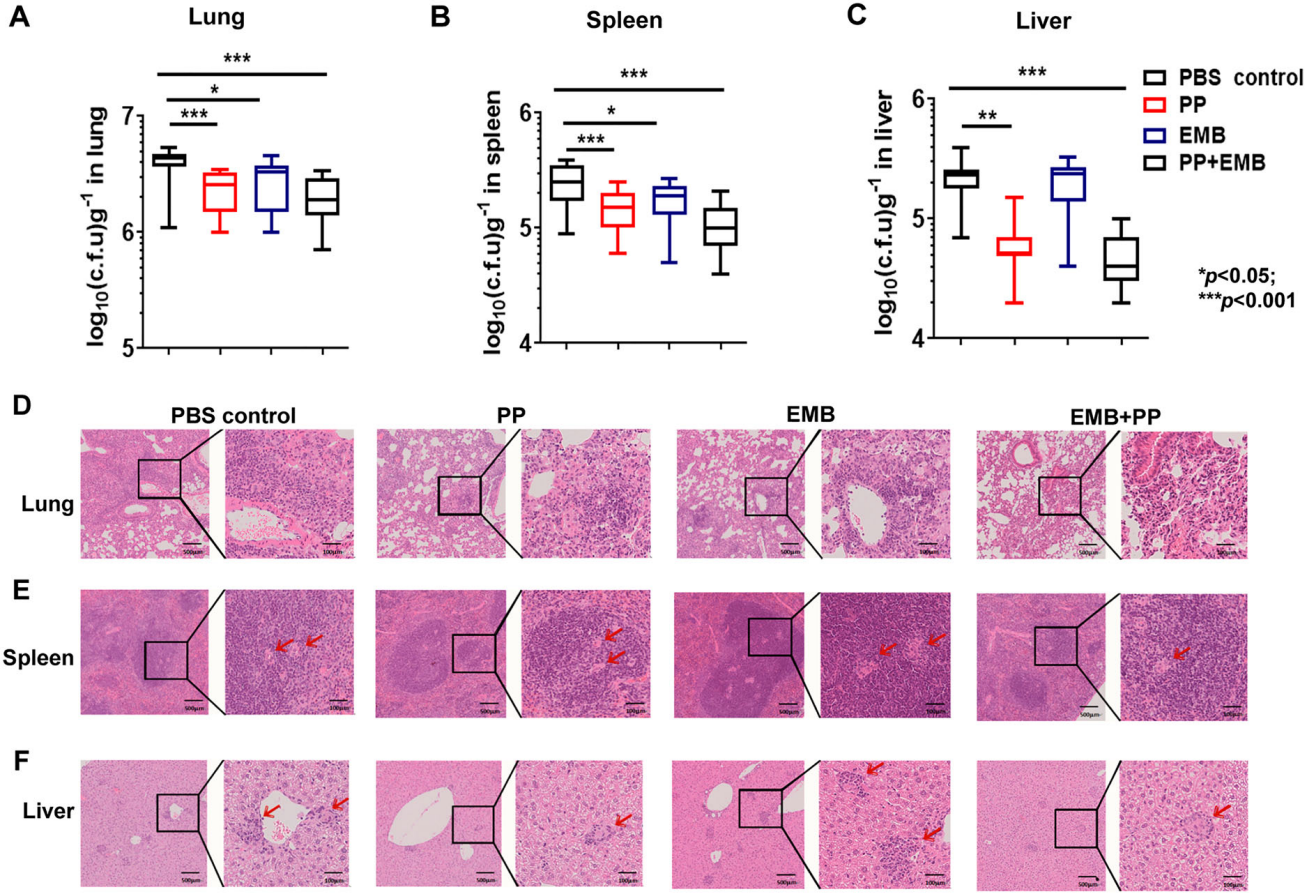
PP inhibits MDR *M. tb* 8462 growth in mice. The mice were infected (*i. v.*) with *M. tb* 8462 (1×10^5^ CFU/mouse), and subjected to treatment with PP, EBM or PP in combination with EBM. After 6 weeks of therapy treatment, the bacterial loads in the lungs (A), spleens (B) and livers (C) were assessed. The pathological sections of lungs, spleens and livers were examined with H&E. The data in (A)–(C) are shown as the means ± SD (*n* = 6). The arrows in (D)–(F) indicate the pulmonary granulomas (D), disorganization of WP and RP in spleen (E), or liver lesions (F).

In mouse model of *M. tb* 8462 infection, the mice were *i.v.* infected with *M. tb* 8462 on week −1 and treated with drugs on week 0. After 6 weeks of the treatment, compared with PBS control group, PP treatment significantly reduced the bacterial CFUs in lung, spleen and liver tissues, especially in liver, and had better anti-XDR *M. tb* 8462 effects than EMB did *in vivo* (Figure 7(A–C)). Moreover, PP plus EMB treatment decreased *M. tb* 8462 growth in the lung, spleen and liver tissues than EMB group, indicating there was a slight synergic effect between PP and EMB (Figure 7(A–C)). Histological analysis also revealed that PP treatment effectively reduced lesion and inflammation in lung, introduced lymphoid hyperplasia in spleens and alleviated hepatocyte swelling, and displayed better protective effects than EMB did (Figure 7(D–F)).

## Discussion

Studies have shown that in the last 40 years, only three new anti-TB drugs have been approved, namely Bedaquiline, Delamanid, and pretomanid worldwide [17–19]. The burden of TB and drug-resistant TB (DR-TB) continues to increase globally. 9% of DR-TB TB patients will develop MDR-TB even XDR-TB [2]. Both MDR-TB and XDR-TB are dangerous medical conditions that can affect patient outcomes, particularly mortality and TB control. Limited therapeutic drug options make the treatment of MDR-TB and XDR-TB difficult [20]. SOSA provides an attractive strategy, by which new potential pharmacological activity of an old approved drug may be identified. Although some new anti-malarial, antibacterial and antiviral drugs have been developed by SOSA [7–10,14,21–23], there is rarely reports about screening and identifying anti-*M.tb* drugs through this approach. In the present study, we used SOSA approach to screen out a new anti-*M. tb* drug, PP. We identified PP had remarkable inhibitory effects on *M.tb* and drug-resistant *M. tb in vitro* and *in vivo*.

*M. smegmatis*, BCG and *M. tb* H37Ra have a similar phenotype and characteristic to virulent *M. tb*. These mycobacteria have been commonly employed for screening anti-mycobacterial drugs [16,24]. In the current study, six candidate drugs (PP, BZK, meclizine hydrochloride, suloctidil, doxorubicin and teniposide) were selected from a library of known 1280 drugs and compounds using *M. smegmatis* as a target for the initial rapid screening. PP and BZK out of the six candidate drugs were identified to be capable of remarkably inhibiting the growth of BCG and *M.tb* H37Ra in a dose-dependent manner. PP had the lower MIC_99_ than BZK had, indicating that PP had the best anti-*Mycobacterium* effect among these drugs (Figure 2(B) and Table 2). Similarly, Kathryn et al used a medium-throughput assay using the Alamar Blue reagent to screen novel inhibitors of *M. tb* from a library of known 1514 drugs and also identified that five compounds including PP (Primaquine, Pentamide, Nialamide, PP, Thiostrepton and Isoniazid) were active against intracellular *M. tb* in a macrophage model of infection *in vitro* activity against *M.tb* H37Rv [25].

PP is a cyanine dye and is a classical anthelminthic officially approved by the FDA in 1955 (NDA-9582) [26]. When taken orally, the dose for human commonly was 5 mg/kg/day, up to 350 mg, it is safe even at high doses, and its dose could be up to 35 mg/kg/day for 3–5 days [27,28]. As a drug against pinworm, the main role of PP is to interfere with the respiratory system of worms, inhibit the uptake of oxygen, increase the anaerobic glycolysis of sugar and impede the absorption of glucose, which causes the parasite to die, but does not kill the eggs [24,26,29]. This drug has been replaced by a more effective broad-spectrum anthelmintics in United States, but it is still employed in Europe and Japan [26]. It has been reported that PP inhibited the nicotinamide adenine dinucleotide (reduced form, NADH) in the respiratory chain and thus play an anti-tumor effect [30,31]. We speculated that PP inhibited the growth of *M. tb* by suppressing NAD+/NADH conversion within the bacteria, but this requires further experimental validation. Recently, some studies reported that PP had pharmacological activity to inhibit the growth of *Bartonella henselae* infection [32] and coronaviruses replication *in vitro* [33,34]. PP has also been reported to have anti-tumor activity [28,34–37]. MIC values of *M. tb* H37Rv and *M. tb* 8462 are similar, however, in the murine model, PP activity against *M. tb* H37Rv is greater than against *M. tb* 8462 (Table 3 and Figure 7), possibly due to the complexity of *M. tb* 8462 pathogenesis *in vivo*. Further study is required to explore the mechanism of PP anti-mycobacterial activity.

Studies have shown that INH inhibits the growth of *M. tb* by suppressing the synthesis of mycolic acid, thus achieving the goal of treating TB [38]. RIF inhibits the growth of *M. tb* by binding the β subunit of RNA polymerase and suppressing transcription [39]. Therefore, the mechanism of PP inhibiting mycobacterial growth might be different from the antimycobacterial mechanisms mediated by INH and RIF. Consequently, PP showed its strong inhibitory effects on the growth of RIF-resistant, INH-resistant and even MDR-resistant *M.tb* clinical isolates *in vitro* (Figure 4(B–D)).

We hardly observed the *M. tb* H37Rv, *M. tb* 94789 and *M. tb* 8462 strains in the mouse lung tissue sections with Ziehl–Neelsen acid-fast stain, which is possibly due to less than 10^6^ CFUs/g tissues during *M. tb* infection. Therefore, bacterial loads in the organs of infected mice were assessed by plating organ homogenates into medium and were determined by CFU counting (Figures 5–7). Although the amount of PP used in a murine model (0.5-1.5 mg/kg/day) is much lower than the amount of RIF and EMB used (20 and 100 mg/kg/day respectively), PP significantly inhibited the *M. tb* infection *in vivo* compared with PBS control groups (Figures 5–7), strongly demonstrating that PP had antimycobacterial activity *in vivo*. However without evaluation of antimycobacterial activity of PP at various concentrations *in vivo*, it is difficult to conclude which drug had the strongest anti-TB effects and whether PP had synergic effects with RIF (or EMB). Therefore, anti-TB function of various concentrations of PP should be investigated in a murine *M. tb* infection model in future studies.

In summary, three major observations were made in this study. First, PP was screened out as an anti-TB agent by SOSA and has remarkable inhibitory effects on the growth of multiple *M. tb* strains (including *M. tb* H37Rv, H37Ra, BCG) *in vitro*. Second, PP has anti-TB effects *in vivo*. Third, PP can also inhibit drug-resistant *M. tb in vitro* and *in vivo*. Our study may have far-reaching implications for the potential application of PP for the treatment of TB in the future.

## Supplementary Material

Supplemental Material
