# Supplementary material for: Identification of pyrvinium pamoate as an anti-tuberculosis agent in vitro and in vivo by SOSA approach amongst known drugs
Source: Emerg Microbes Infect. 2020 Feb 4;9(1):302–12. doi: 10.1080/22221751.2020.1720527 (PMC7034053; doi:10.1080/22221751.2020.1720527)
Supplement: Supplemental Material [file TEMI_A_1720527_SM8149.zip › Supplemental_table_1_final.docx]

**STable 1. Nine drugs selected for inhibiting *M. smegmatis growth* in the initial round of selection**

| Drugs | Reported pharmacological activities |
| --- | --- |
| Suloctidil | Antipyretic and analgesic |
| Teniposide | Commonly used in the treatment of primary or secondary central nervous system malignancies |
| Isoproterenol hydrochloride | Treat cardiogenic or septic shock and complete atrioventricular block, cardiac arrest |
| Daunorubicin | The anthracycline family of medication. Partly block the function of topoisomerase II |
| PP | Enterobiasis treatment |
| Adiphenine hydrochloride | Relieve smooth muscle spasm, relieve visceral cramps, improve circulation and inhibit glandular secretion |
| BZK | Contraceptives |
| Doxorubicin | Anti-tumor |
| Meclizine hydrochloride | Prevent vomiting caused by pregnancy and radiotherapy |
