# Supplementary material for: Identification of pyrvinium pamoate as an anti-tuberculosis agent in vitro and in vivo by SOSA approach amongst known drugs
Source: Emerg Microbes Infect. 2020 Feb 4;9(1):302–12. doi: 10.1080/22221751.2020.1720527 (PMC7034053; doi:10.1080/22221751.2020.1720527)
Supplement: Supplemental Material [file TEMI_A_1720527_SM8149.zip › Supplemental_table_2_final.docx]

**STable 2.** **MIC_99_ values of PP against *M. tb* H37Rv, MDR *M. tb* 94789 and XDR *M. tb* 8462**

|  | ***M. tb* H37Rv** | ***M. tb* 94789** | ***M. tb* 8462** |
| --- | --- | --- | --- |
| **STR** (4 μg/mL) | Sensitive | Resistant | Resistant |
| **INH** (0.2 μg/mL) | Sensitive | Resistant | Resistant |
| **RIF** (5 μg/mL) | Sensitive | Resistant | Resistant |
| **PP** MIC | 1.5 μg/mL | 3 μg/mL | 0.75 μg/mL |
