# Supplementary figures and images for: Identification of pyrvinium pamoate as an anti-tuberculosis agent in vitro and in vivo by SOSA approach amongst known drugs
Source: Emerg Microbes Infect. 2020 Feb 4;9(1):302–12. doi: 10.1080/22221751.2020.1720527 (PMC7034053; doi:10.1080/22221751.2020.1720527)

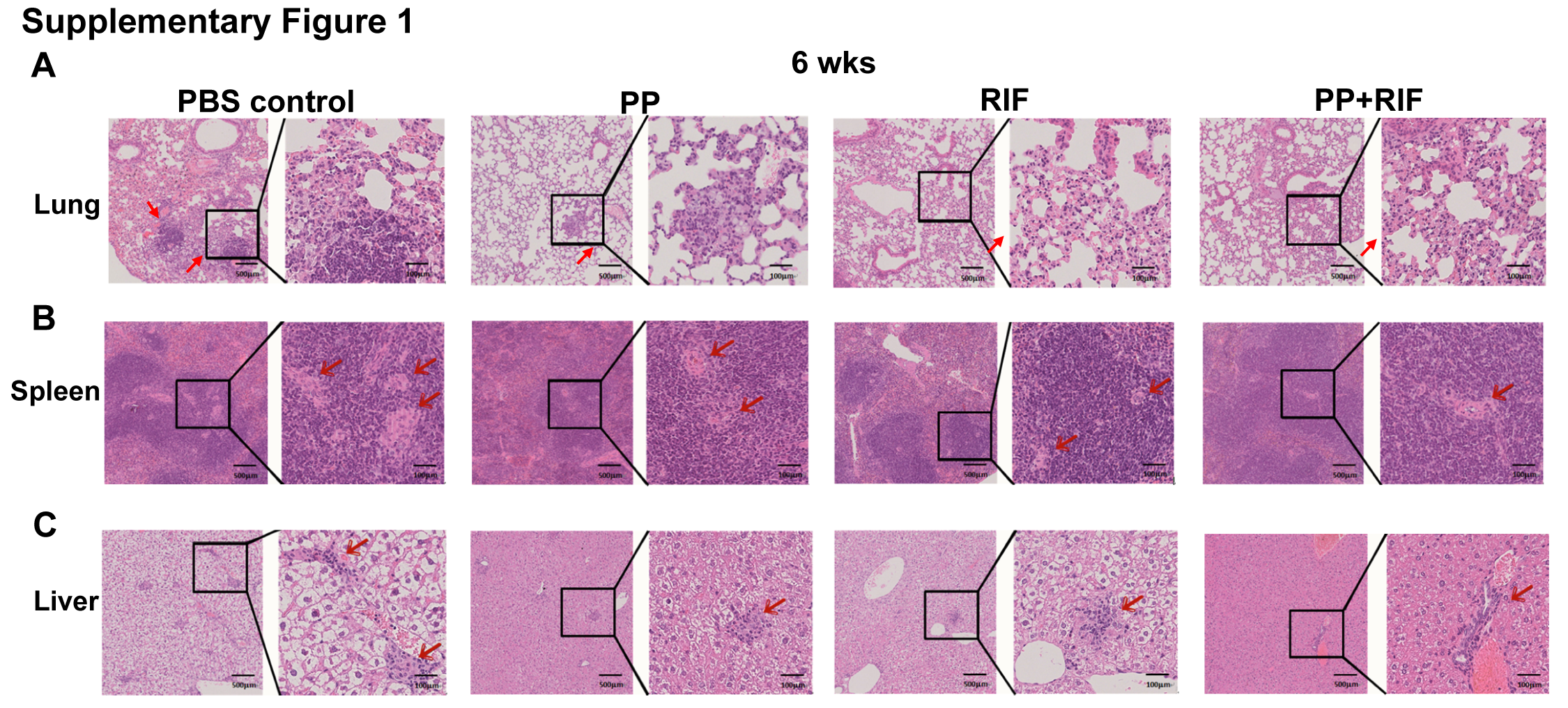

Supplement: Supplemental Material [file TEMI_A_1720527_SM8149.zip › Fig_S1(1)_final.tif]
